# Gender bias in assessing narcissistic personality: Exploring the utility of the ICD‐11 dimensional model

**DOI:** 10.1111/bjc.12503

**Published:** 2024-09-11

**Authors:** A. Green, N. J. S. Day, C. M. Hart, B. F. S. Grenyer, B. Bach

**Affiliations:** ^1^ Department of Psychology City University of London London UK; ^2^ School of Psychology University of Wollongong Wollongong New South Wales Australia; ^3^ Department of Psychology University of Southampton Southampton UK; ^4^ Psychiatric Research Unit, Center for Personality Disorder Research Mental Health Services Region Zealand Denmark; ^5^ Department of Psychology University of Copenhagen Copenhagen Denmark

**Keywords:** diagnosis, female narcissism, gender bias, grandiose and vulnerable narcissism, pathological narcissism

## Abstract

**Objectives:**

Narcissistic personality disorder as captured in categorical diagnostic systems (e.g., DSM‐5) emphasizes grandiose features more associated with masculine norms and under‐emphasizes vulnerable features more associated with femininity. This poses significant implications in diagnostic outcome and clinical treatment in women with narcissistic preoccupations. Research finds that clinicians using the DSM‐5 categorical system tend to diagnose vulnerable narcissism in women as other ‘feminized’ personality disorders (e.g., borderline), but no research has explored gender differences in narcissism using the new ICD‐11 dimensional framework for personality disorders. This study investigated the clinical utility of the ICD‐11 approach in capturing gender differences in narcissistic presentations.

**Methods:**

Adopting an online vignette‐based study, mental health clinicians (*N* = 157; 71.3% female) completed ratings of ICD‐11 personality disorder severity and trait domains for two cases reflecting ‘grandiose’ and ‘vulnerable’ narcissism in hypothetical male or female patients.

**Results:**

The results showed that ratings of core impairments in personality functioning and overall severity were consistent irrespective of patient or clinician gender, contrasting prior research using categorical models.

**Conclusion:**

While some differences were observed in trait domain (e.g., negative affectivity) between patient gender, these results suggest the clinical utility of the ICD‐11 model as emphasizing elements of personality functioning in the process of assessment and diagnosis, therefore potentially being less susceptible to influences of gender stereotype in aiding clinical conceptualization.

## INTRODUCTION

Narcissistic personality disorder (NPD) is a diagnosable personality disorder marked by grandiose self‐importance, omnipotent fantasy, belief in specialness, admiration seeking, entitlement, envy and interpersonal difficulties such as exploitativeness, lacking empathy and antagonism (American Psychiatric Association, 2013). It is known to be a challenging and severe disorder often involving complex transference and countertransference constellations that can complicate treatment trajectory and outcome (Crisp & Gabbard, 2020; Diamond et al., 2021). However, while these criteria reflect an overt, grandiose type presentation, a more fragile and vulnerable presentation of narcissism has also been identified (Cain et al., 2008; Day et al., 2020; Levy, 2012; Russ et al., 2008), which encompasses a display of overt shyness, hypersensitivity, shame and negative affect. The clinical literature has increasingly stressed that narcissistic individuals vacillate between grandiose and vulnerable expressions (Gore & Widiger, 2016; Oltmanns & Widiger, 2018). In other words, the grandiose type may commonly display behavioural traits reflecting overt grandiosity, entitlement, and exhibitionism, yet they will experience extreme insecurity, depletion and self‐loathing in the face of ego‐threat or failure. Conversely, the vulnerable type may present themselves as timid and hypersensitive, but over time reveal exhibitionistic and grandiose fantasies (Levy, 2012; Pincus et al., 2014). In recognition of this, clinical researchers have concluded that narcissistic patients are best differentiated based on their relative levels of grandiosity and vulnerability, which can be expressed both overtly and covertly, rather than by categorical distinctions (Pincus & Lukowitsky, 2010). The umbrella term ‘pathological narcissism’ is used to reflect this variable presentation of grandiosity and vulnerability, while retaining the fundamentally narcissistic style (Kealy & Rasmussen, 2011; Pincus & Lukowitsky, 2010). The presentation of narcissism (grandiose, vulnerable or a fluctuation between the two) can greatly influence the therapeutic situation, regarding assessment (Pincus et al., 2016), gender (Green et al., 2023), countertransference (Tanzilli & Gualco, 2020) and treatment tactics (Ellison et al., 2013; Pincus et al., 2014).

## THE ICD‐11 DIMENSIONAL MODEL OF PERSONALITY

This year, the *International Classification of Disease* (ICD‐11; World Health Organization, 2024) has officially implemented a dimensional approach to conceptualizing personality pathology, utilizing a dual ‘severity’ and ‘trait domain’ system to classify personality disorders (Bach & First, 2018). Severity in personality dysfunction relate to core impairments in ‘self’ (e.g., identity, self‐worth, capacity for self‐direction) and ‘interpersonal’ (e.g., intimacy, mutuality, conflict management) domains. This system is similar to early dimensional models such as personality ‘organization’ (Kernberg, 1967), and has seen repeated empirical examination and affirmation regarding a common core elements of personality dysfunction (Ringwald et al., 2019; Sharp et al., 2015; Williams et al., 2018; Wright et al., 2016). While evidence is still emerging for the utility of the new ICD‐11 classification system, early indicators highlight the improved clinical utility and appropriateness of the new model (Tracy et al., 2021). Such improvements include the sound empirical foundation of the severity and trait domain format of which the model is built around, its ability to facilitate enhanced clinical decision making around issues of prognosis, risk and intensity of required psychotherapy, as well as furthering anti‐stigma efforts by focusing on common issues of humanity (i.e., identity and relationships) as opposed to an alienating disease model framework (Bach et al., 2022; Herpertz et al., 2022; Mulder & Tyrer, 2023; Stricker et al., 2024).

The ICD‐11 severity of personality dysfunction is indexed with five levels of impairment, from ‘None’ through to ‘Severe Personality Disorder’, with a minimum of ‘Mild Personality Disorder’ needed for a diagnosis. Narcissistic personality in particular appears well suited to be conceptualized according to a dimension of severity (Russ et al., 2008), as the literature on narcissism has alternatively described ‘adaptive’ high functioning narcissism that is (at best) related to agentic striving and leadership success (Ackerman et al., 2011) or (at worst) constituting defensive self‐regulatory processes (Hörz‐Sagstetter et al., 2018; Kaufman et al., 2018). At the opposite end of the spectrum, authors have also described a severe and extreme version termed ‘malignant narcissism’ which blends features of paranoia and sadism resulting in serious moral deficits and violence (Kernberg, 2007; Lenzenweger et al., 2018). Regarding personality trait domains, the ICD‐11 utilize maladaptive variants of the five‐factor model of personality (McCrae & Costa, 1997): ‘Negative Affectivity’, ‘Detachment’, ‘Disinhibition’, ‘Dissociality’ and ‘Anankastia’. In terms of trait domains of relevance for narcissism, dissociality has most consistently been associated (Simon et al., 2023) as this includes instances of entitlement, grandiosity and interpersonal dysfunction which correspond most closely to the traditional categorical criteria of narcissism. Negative affectivity has also been highlighted as an important consideration covering instances of affective instability, low self‐esteem and pessimism (Bach & First, 2018), as this enables capturing the more vulnerable configuration of narcissism (Bach & Tracy, 2022; Pincus et al., 2016). Anankastia has also been indicated (Fjermestad‐Noll et al., 2020; Simon et al., 2023), corresponding to a ‘narcissistic perfectionism’ (Day et al., 2020; Nealis et al., 2015) that includes hypersensitivity to critiques from others and/or expecting others to maintain unrealistic standards and corresponding subsequent devaluation.

## GENDER DIFFERENCES AND GENDER BIAS IN NARCISSISTIC PERSONALITY

Prevalence of NPD diagnoses differ significantly between men (75%) and women (25%) in clinical populations (American Psychiatric Association, 2013) and in the general population (7.7% in men compared to 4.4% in women; Stinson et al., 2008). Such gender differences are argued to reflect true sex differences “driven by men's heightened sense of entitlement and authority” (Grijalva et al., 2014, p. 284). However, these findings are contingent on the validity of the narcissism construct being utilized to establish such differences—namely DSM‐IV criteria and the Narcissistic Personality Inventory—which strongly favour narcissistic grandiosity (Cain et al., 2008). It is also possible that observed gender differences in narcissistic presentation reflect the influence of socially prescribed cultural norms of masculinity and femininity that shapes expression (Green et al., 2022). As such, grandiose traits which overlap with masculine features (e.g., entitlement, superiority, assertiveness) tend to characterize male narcissism, whereas vulnerable traits which closely associates with femininity (e.g., emotional vulnerability, inhibition, low self‐esteem) tend to characterize female narcissism. However, the literature has traditionally treated narcissism as exclusively a male pathology, whereas narcissism in women has been overlooked (Green et al., 2022). As the symptomatology of the narcissistic disorder is biased towards masculine tendencies, this introduces gender bias in assumptions about the disorder which has implications for assessment, diagnosis and treatment, as clinicians may underdiagnose or misdiagnose women's manifestations of narcissistic psychopathology as other ‘feminized’ disorders such as borderline, histrionic or dependent personality (Anderson et al., 2001; Euler et al., 2018). Indeed, in a vignette‐based study, Green et al. (2023) report that when the gender of hypothetical patients with vulnerable narcissistic features are randomized, clinicians are significantly more likely to attribute a borderline personality disorder diagnosis to female cases, whereas male patients are diagnosed with ‘other’ conditions (e.g., social anxiety, depression). Similarly, hypothetical male patients with grandiose features were more likely to be attributed as narcissistic personality disorder or antisocial personality disorder. These results support the notion that personality disorder categories are laden with gender bias that influences clinician conceptualization.

## THE CURRENT STUDY

Given the gendered connotations that exist within the categorical construct of narcissism, research is needed to examine whether new dimensional models of personality (e.g., DSM‐5‐AMPD, ICD‐11) can lead to the provision of more accurate diagnoses, less influenced by gender bias. In particular, the wholesale removal of diagnostic labels the ICD‐11 classification system represents a unique opportunity to explore clinician's conceptualization of narcissistic functioning in the absence of priming diagnostic labels that may infer gender stereotype and bias. As such, this study aims to examine how prototypically narcissistic hypothetical patients are understood within the ICD‐11 framework, to examine the influence of gender on clinician ratings of personality severity and trait domains. This is the first study to systematically examine gender differences in narcissism through the lens of the ICD‐11.

## METHOD

### Participants

Participants needed to be qualified and be actively involved in the provision of mental health services to people with a personality disorder (e.g., clinicians, psychologists, psychiatrists, mental health nurses, social workers, etc.) to take part in the study. Participants were recruited using a snowball sampling methodology via advertisements posted to universities and local health facilities. Participants were entered into a prize draw to win a gift voucher for participating in the research. From an initial sample pool (*N* = 334), 175 participants were excluded for partial completion; we required data for both vignettes to be completed. Two participants were excluded for not identifying as male or female. The final data set comprised of 157 participants, including 112 females (71.3%) and 45 males (28.7%). Table 1 displays the demographics for the sample.

**TABLE 1:** Study demographics.

	All participants (*N* = 157)	Male clinicians (*n* = 45)	Female clinicians (*n* = 112)
Ethnicity (%)			
White	129 (82.2)	38 (84.4)	91 (81.3)
African	2 (1.3)	1 (2.2)	1 (0.9)
South or East Asian	9 (5.7)	1 (2.2)	8 (7.1)
Latino or Hispanic	1 (0.6)	1 (2.2)	‐
Middle Eastern	6 (3.8)	3 (6.7)	3 (2.7)
Mixed	7 (4.5)	1 (2.2)	6 (5.4)
Other	3 (1.9)	‐	3 (2.7)
Mean age (SD)	38.15 (11.47)	40.58 (11.54)	37.17 (11.34)
Mean years clinical experience (SD)	11.13 (10.10)	13.88 (10.60)	10.02 (9.72)
Education (%)			
MD	8 (5.1)	4 (8.9)	4 (3.6)
PhD	36 (22.9)	14 (31.1)	22 (19.6)
Master's degree or equivalent	70 (44.6)	13 (28.9)	57 (50.9)
Honours degree or equivalent	28 (17.8)	9 (20.0)	19 (17.0)
Bachelor's degree or equivalent	10 (6.4)	4 (8.9)	6 (5.4)
Other	5 (3.2)	1 (2.2)	4 (3.6)
Title (%)			
Provisional/trainee psychologist	49 (31.2)	8 (17.8)	41 (36.6)
Registered/chartered psychologist	20 (12.7)	5 (11.1)	15 (13.4)
Clinical psychologist	40 (25.5)	15 (33.3)	25 (22.3)
Psychiatry registrar	1 (0.6)	1 (2.2)	‐
Psychiatrist	15 (9.6)	10 (22.2)	5 (4.5)
Other	32 (20.4)	6 (13.3)	26 (23.2)
PD expertise (%)			
Minimal	12 (8.3)	1 (2.3)	11 (11.0)
Developing	43 (29.9)	10 (22.7)	33 (33.0)
Sound	38 (26.4)	9 (20.5)	29 (29.0)
Advanced	28 (19.4)	9 (20.5)	19 (19.0)
Expert	13 (16.0)	15 (34.1)	8 (8.0)
Therapeutic modality (%)			
Skills‐based	58 (37.2)	11 (24.4)	47 (42.3)
Dynamic theories	30 (19.2)	14 (31.1)	16 (14.4)
Mixed approach	66 (42.3)	18 (40.0)	48 (43.2)
None	2 (1.3)	2 (4.4)	0 (0)

### Measures

#### Clinical case vignettes

Participants were presented with clinical case vignettes of hypothetical male or female patients presenting prototypical expressions of grandiose narcissism *and* vulnerable narcissism. These vignettes were constructed by Kealy et al. (2017) as informed by an expert review of the central features of narcissistic phenotypic expression (Cain et al., 2008). The grandiose and vulnerable vignettes are designed to be equivalent in terms of displaying clinical indicators of personality dysfunction and have been previously used in exploratory research (Green et al., 2023).

#### ICD‐11 aspects of severity

Clinical vignettes were scored using eight items modified from the Personality Disorder Severity ICD‐11 Scale (PDS‐ICD‐11; Bach et al., 2022) to capture the level of severity in aspects of personality functioning as conceptualized in the ICD‐11 (identity, self‐worth, self‐perception, goals, relationship interest, empathy, mutuality, conflict management). The PDS‐ICD‐11 has been shown to be a valid and reliable measure of personality severity (Bach et al., 2022). Items are rated on a 5‐point scale (i.e., 2–1–0–1–2), with the centre point (‘0’) representing healthy functioning and outer points (‘2’) representing impairment in functioning. Each end of the scale reflects opposing extremes of impairment (e.g., for the ‘identity’ aspect the extreme scores capture either ‘absent’ or ‘rigid’, respectively). We created an overall severity index by averaging across these eight items. Internal consistency within the present sample was acceptable (*α* = .72).

#### Global personality disorder severity

One question was used to assess overall personality disorder severity (“At what degree of overall personality disorder severity would you rate [the patient]?”). Clinicians responded to this question on a scale from 0 (*no personality disorder*) to 4 (*severe personality disorder*), using the severity classifications in the ICD‐11. Scores of two (*mild personality disorder*) and above indicated a personality disorder (World Health Organization, 2024).

#### ICD‐11 personality traits and facets

Clinicians were asked to endorse the personality trait domains that were relevant to the vignettes from those outlined in the ICD‐11 (negative affectivity, detachment, dissociality, disinhibition, anankastia; 0 = *not selected*, 1 = *selected*).

#### Borderline pattern specifier

The borderline pattern specifier is included in the ICD‐11 to identify individuals who may respond to certain psychotherapeutic treatments. Clinicians were presented criteria of borderline personality disorder (BPD) as they appear in the DSM‐5 (American Psychiatric Association, 2013) and asked to indicate whether the patient in the vignette displayed five or more of these criteria. Endorsement of the borderline pattern specifier was scored as 1 (vs. 0 for lack of endorsement).

### Procedure

The study was advertised as ‘Personality in the ICD‐11 Framework’ via e‐mails sent to clinical psychology committees and universities to distribute the study to a broader sample of psychologists working in the provision of mental health services. In the invitation email sent to prospective participants, there was no mention that the study would be focusing on narcissistic personality so participants were not aware of the specific interest of the study that may bias their ratings. After giving informed consent, participants completed demographic questions and were randomly assigned either two female vignettes or two male vignettes to avoid priming participants to gender bias. The two vignettes depicted a hypothetical patient exhibiting symptoms of vulnerable narcissism and another depicting grandiose narcissistic symptomatology. After reading each vignette, participants indicated the severity of symptoms and endorsed the relevant personality trait domains corresponding to that vignette. After rating both vignettes, participants were debriefed and thanked for their participation.

## RESULTS

Participants completed severity and trait domain ratings of vulnerable and grandiose patients, thus we had nested data; multiple measures nested within individuals. To account for violations of observation independence, we used Generalized Estimating Equations (GEEs). GEEs can handle both continuous and binary outcome variables adopted in this study (Huang, 2022). All analyses were conducted using SPSS Version 28.

Descriptive data broken down by clinician gender, patient gender and narcissistic symptomology are found in Table 2. All variables were normally distributed. Due to the large number of analyses conducted, we adopted a stricter significance level of *p* < .01 to reduce the chance of encountering a Type I error.

**TABLE 2:** Mean values and standard deviations (for continuous data) and frequencies and percentages (for binary outcomes) of key variables.

	All participants (*N* = 157)				Male clinicians (*n* = 45)				Female clinicians (*n* = 112)			
	Vulnerable male (*n* = 77)	Grandiose male (*n* = 77)	Vulnerable female (*n* = 80)	Grandiose female (*n* = 80)	Vulnerable male (*n* = 26)	Grandiose male (*n* = 26)	Vulnerable female (*n* = 19)	Grandiose female (*n* = 19)	Vulnerable male (*n* = 51)	Grandiose male (*n* = 51)	Vulnerable female (*n* = 61)	Grandiose female (*n* = 61)
Personality domains (M SD)												
Identity	1.42 (0.62)	1.44 (0.64)	1.47 (0.55)	1.39 (0.61)	1.27 (0.67)	1.46 (0.65)	1.63 (0.50)	1.21 (0.54)	1.49 (0.58)	1.43 (0.64)	1.43 (0.56)	1.44 (0.62)
Self‐esteem	1.49 (0.58)	1.82 (0.39)	1.59 (0.54)	1.81 (0.45)	1.50 (0.58)	1.81 (.40)	1.68 (0.58)	1.89 (0.32)	1.49 (0.58)	1.82 (0.39)	1.56 (0.53)	1.79 (0.49)
Self‐worth	1.00 (0.56)	1.68 (0.52)	1.19 (0.53)	1.65 (0.53)	1.08 (0.63)	1.69 (0.55)	1.05 (0.52)	1.74 (0.45)	0.96 (0.53)	1.67 (0.52)	1.23 (0.53)	1.62 (0.55)
Self‐direction	0.83 (0.68)	1.23 (0.72)	0.88 (0.68)	1.20 (0.68)	0.88 (0.77)	1.19 (0.75)	0.68 (0.67)	1.11 (0.81)	0.80 (0.63)	1.25 (0.72)	0.93 (0.68)	1.23 (0.64)
Relationship interest	0.92 (0.64)	0.91 (0.67)	0.99 (0.65)	0.83 (0.61)	0.88 (0.59)	0.92 (0.74)	0.84 (0.69)	0.68 (0.58)	0.94 (0.68)	0.90 (0.64)	1.03 (0.63)	0.87 (0.62)
Empathy	1.44 (0.62)	1.52 (0.58)	1.54 (0.55)	1.41 (0.59)	1.46 (0.71)	1.58 (0.58)	1.42 (0.61)	1.58 (0.51)	1.43 (0.58)	1.49 (0.58)	1.57 (0.53)	1.36 (0.61)
Mutuality	1.12 (0.65)	1.71 (0.48)	1.29 (0.64)	1.61 (0.56)	1.00 (0.75)	1.73 (0.53)	1.16 (0.77)	1.89 (0.32)	1.18 (0.59)	1.71 (0.46)	1.33 (0.60)	1.52 (0.60)
Conflict management	1.34 (0.60)	1.35 (0.60)	1.45 (0.65)	1.35 (0.62)	1.35 (0.63)	1.31 (0.55)	1.37 (0.68)	1.53 (0.51)	1.33 (0.59)	1.37 (0.63)	1.48 (0.65)	1.30 (0.64)
Overall severity (M SD)	1.19 (0.38)	1.46 (0.33)	1.30 (0.35)	1.41 (0.31)	1.18 (0.45)	1.46 (0.43)	1.23 (0.37)	1.45 (0.25)	1.20 (0.34)	1.46 (0.28)	1.32 (0.34)	1.39 (0.33)
Global severity (M SD)	2.13 (0.99)	2.82 (0.87)	2.25 (0.92)	2.68 (0.91)	2.38 (1.06)	2.96 (0.87)	2.58 (0.84)	3.11 (0.74)	2.00 (0.94)	2.75 (0.87)	2.15 (0.93)	2.54 (0.92)
Above PD threshold (frequency %)	56 (72.7)	70 (90.9)	63 (78.7)	71 (88.7)	22 (84.6)	24 (92.3)	18 (94.7)	19 (100)	34 (66.7)	46 (90.2)	45 (73.8)	52 (85.2)
Below PD threshold (frequency %)	21 (27.3)	7 (9.1)	17 (21.3)	9 (11.3)	4 (15.4)	2 (7.7)	1 (5.3)	0 (0)	17 (33.3)	5 (9.8)	16 (26.2)	9 (14.8)
Trait Domains (frequency %)												
Negative Affectivity	74 (96.1)	30 (39.0)	75 (93.8)	22 (27.5)	24 (92.3)	11 (42.3)	19 (100)	4 (21.1)	50 (98.0)	19 (37.3)	56 (91.8)	18 (29.5)
Detachment	56 (72.7)	30 (39.0)	59 (73.8)	25 (31.3)	19 (73.1)	9 (34.6)	12 (63.2)	5 (26.3)	37 (72.5)	21 (41.2)	47 (77.0)	20 (32.8)
Dissociality	12 (15.6)	72 (93.5)	8 (10.0)	72 (90.0)	5 (19.2)	25 (96.2)	2 (10.5)	17 (89.5)	7 (13.7)	47 (92.2)	6 (9.8)	55 (90.2)
Disinhibition	5 (6.5)	32 (41.6)	2 (2.5)	38 (47.5)	3 (11.5)	10 (38.5)	1 (5.3)	9 (47.4)	2 (3.9)	22 (43.1)	1 (1.6)	29 (47.5)
Anankastia	43 (55.8)	46 (59.7)	40 (50.0)	45 (56.3)	12 (46.2)	12 (46.2)	9 (47.4)	8 (42.1)	31 (60.8)	34 (66.7)	31 (50.8)	37 (60.7)
Borderline specifier (frequency %)												
Met	21 (27.3)	11 (14.3)	25 (31.3)	8 (10.0)	8 (30.8)	4 (15.4)	5 (26.3)	0 (0)	13 (25.5)	7 (13.7)	20 (32.8)	8 (13.1)
Unmet	56 (72.7)	66 (85.7)	55 (68.8)	72 (90.0)	18 (69.2)	22 (84.6)	14 (73.7)	19 (100)	38 (74.5)	44 (86.3)	41 (67.2)	53 (86.9)

### Severity

GEE analyses were conducted with overall severity (a composite of eight PDS items) as the dependent variable, and with two between‐subject predictors; clinician gender (male = 0, female = 1), patient gender (male = 0, female = 1), and one within‐subject predictor; narcissism (vulnerable = 0, grandiose = 1). We included length of time as a clinician (centred) as a covariate. The test of model effects revealed only a significant effect of narcissism (see Table 3); clinicians rated patient symptoms as more severe when presented with a patient with grandiose (*M* = 1.44, SE = .03) than vulnerable narcissism (*M* = 1.23, SE = .03). For a breakdown of each of the severity items, see Data S1.

**TABLE 3:** Test of model effects on overall severity and global severity scores.

	Overall severity			Global severity		
Variables	Wald *χ* ^2^	Df	*p*	Wald *χ* ^2^	Df	*p*
(Intercept)	73,102,389.33	1	<.001	1482.90	1	<.001
Clinician gender	.13	1	.719	6.45	1	.011
Patient gender	.16	1	.690	.13	1	.714
Narcissism	41.18	1	<.001	63.00	1	<.001
Length of clinical practice	.88	1	.349	6.05	1	.014
Clinician gender × patient gender	.01	1	.940	.39	1	.531
Clinician gender × narcissism	2.00	1	.158	.02	1	.901
Patient gender × narcissism	3.45	1	.063	2.03	1	.154
Clinician gender × patient gender × narcissism	.87	1	.351	1.14	1	.286

We also tested an alternative measure of severity using the global personality disorder severity scale, with higher scores indicating more severe symptoms. GEE analyses were conducted with global severity as the dependent variable, and with two between‐subject predictors; clinician gender (male = 0, female = 1), patient gender (male = 0, female = 1), and one within‐subject predictor; narcissism (vulnerable = 0, grandiose = 1). We included length of time as a clinician (centred) as a covariate. GEE results revealed a significant effect of narcissism (see Table 3); as above, clinicians rated patient symptoms as being more severe when presented with a patient with grandiose (*M* = 2.82, SE = .07) than vulnerable (*M* = 2.26, SE = .08) narcissism.

### Trait domains

We tested a series of GEEs on five trait domains (see Table 4). In each case, the trait domain was the dependent variable (not selected = 0, selected = 1), along with two between‐subject predictors; clinician gender (male = 0, female = 1), patient gender (male = 0, female = 1) and one within‐subject predictor; narcissism (vulnerable = 0, grandiose = 1). We included length of time as a clinician (centred) as a covariate.

**TABLE 4:** Test of model effects on trait domains.

	Negative affect			Detachment			Dissociality			Disinhibition			Anankastia		
Variables	Wald *χ* ^2^	Df	*p*	Wald *χ* ^2^	Df	*p*	Wald *χ* ^2^	Df	*p*	Wald *χ* ^2^	Df	*p*	Wald *χ* ^2^	Df	*p*
(Intercept)	250.62	1	<.001	.87	1	.351	3.16	1	.076	40.77	1	<.001	1.10	1	.294
Clinician gender	74.53	1	<.001	.71	1	.400	1.10	1	.294	1.69	1	.194	1.76	1	.184
Patient gender	78.78	1	<.001	.60	1	.440	.58	1	.447	.13	1	.716	.01	1	.907
Narcissism	397.33	1	<.001	39.87	1	<.001	82.43	1	<.001	44.12	1	<.001	.44	1	.508
Length of clinical practice	1.58	1	.209	.92	1	.337	2.07	1	.150	.51	1	.476	6.97	1	.008
Clinician gender × patient gender	278.07	1	<.001	.34	1	.561	.10	1	.758	.08	1	.776	.88	1	.349
Clinician gender × narcissism	87.81	1	<.001	.003	1	.959	.12	1	.727	1.66	1	.198	1.04	1	.309
Patient gender × narcissism	126.91	1	<.001	.27	1	.601	.10	1	.755	2.34	1	.126	.18	1	.676
Clinician gender × patient gender × narcissism	^a^	‐		.42	1	.516	.00	1	.998	.06	1	.805	.00	1	.998

^a^ Unable to compute due to numerical problems.

#### Negative affectivity

The test of model effects revealed significant effects of clinician gender; with male clinicians (*M =* 1.00, SE = .00) more likely to rate the patients as displaying negative affect than female clinicians (*M =* .78, SE = .05). There were significant effects of patient gender; with clinicians reporting vignettes featuring female patients (*M =* 1.00, SE = .00) to be more likely to be experiencing negative affect than male patients (*M =* .80, SE = .05). A significant effect of narcissism suggests that clinicians were more likely to attribute negative affect to the patient when they were displaying vulnerable (*M =* 1.00, SE = .00) than grandiose symptoms (*M =* .31, SE = .04).

We used pairwise comparisons to examine the breakdown of the significant clinician gender × patient gender interaction. Male clinicians rating female patients were more likely to attribute negative affect to the patient (*M =* 1.00, SE = .00) than female clinicians rating female patients (*M =* .69, SE = .06). Male clinicians rating female patients were also more likely to attribute negative affect to the patient (*M =* 1.00, SE = .00) than male clinicians rating male patients (*M =* .69, SE = .06).

Pairwise comparisons were also used to break down the significant clinician gender * narcissism interaction. Female clinicians rated patients in grandiose vignettes (*M =* .34, SE = .05) as less likely to display negative affect than patients in vulnerable vignettes (*M =* .96, SE = .02) and as less likely to display negative affect than male clinicians rating vulnerable vignettes (*M =* 1.00, SE = .00). Female clinicians rated patients in vulnerable vignettes (*M =* .96, SE = .02) as more likely to display negative affect than male clinicians rating grandiose vignettes (*M =* .29, SE = .07). Male clinicians rated vulnerable vignettes (*M =* 1.00, SE = .00) as more likely to display negative affect than patients in grandiose vignettes (*M =* .29, SE = .07).

A significant interaction between patient gender and narcissism was also significant and broken down using pairwise comparisons. Female patients in the grandiose vignette (*M =* .24, SE = .06) were less likely to be perceived as having negative affect compared with female patients displaying vulnerable narcissism (*M =* 1.00, SE = .00) and compared with male patients presenting vulnerable narcissism (*M =* .96, SE = .02). Female patients in the vulnerable vignette (*M =* 1.00, SE = .00) were more to be perceived as having negative affect than male patients in the grandiose vignette (*M =* .40, SE = .06). Male patients presenting grandiose narcissism (*M =* .96, SE = .02) were significantly less likely to be perceived as having negative affect compared with male patients presenting with vulnerable narcissism (*M =* .40, SE = .06).

#### Detachment

The test of model effects revealed only a significant effect of narcissism, such that clinicians were more likely to select detachment in relation to patients presenting with vulnerable (*M =* .72, SE = .04) than grandiose (*M =* .34, SE = .04) narcissism profiles.

#### Dissociality

The test of model effects revealed only a significant effect of narcissism, such that clinicians were more likely to select dissociality in relation to patients presenting with grandiose (*M =* .94, SE = .02) than vulnerable (*M =* .13, SE = .03) narcissism profiles.

#### Disinhibition

The test of model effects revealed only a significant effect of narcissism, such that clinicians were more likely to select disinhibition in relation to patients presenting with grandiose (*M =* .47, SE = .05) than vulnerable (*M =* .05, SE = .02) narcissism profiles.

#### Anankastia

The test of model effects only revealed a significant effect of length of clinical practice, such that clinicians were more likely to select anankastia the less time they had been in practice (*B* = −.04, SE = .02, *p* = .008).

### Borderline specifier

We ran a GEE with a Borderline specifier binary‐dependent variable (does not meet criteria = 0, meets criteria = 1), and with two between‐subject predictors; clinician gender (male = 0, female = 1), patient gender (male = 0, female = 1) and one within‐subject predictor; narcissism (vulnerable = 0, grandiose = 1). We included length of time as a clinician (centred) as a covariate. Tests of model effects are shown in Table 5.

**TABLE 5:** Test of model effects on borderline specifier.

Variables	Wald *χ* ^2^	Df	*p*
(Intercept)	451.00	1	<.001
Clinician gender	209.19	1	<.001
Patient gender	231.23	1	<.001
Narcissism	376.90	1	<.001
Length of clinical practice	1.65	1	.199
Clinician gender × patient gender	297.96	1	<.001
Clinician gender × narcissism	295.20	1	<.001
Patient gender × narcissism	444.21	1	<.001
Clinician gender × patient gender × narcissism	^a^		

^a^ Unable to compute due to numerical problems.

The test of model effects revealed significant effects of clinician gender; with female clinicians (*M =* .19, SE = .03) more likely to perceive the patient as meeting borderline criteria than male clinicians (*M =* .00, SE = .00). There were significant effects of patient gender; with male patients (*M =* .20, SE = .04) being more likely to meet borderline specifiers than females patients (*M =* .00, SE = .00). A significant effect of narcissism suggests that clinicians were more likely to perceive patients displaying vulnerable (*M =* .29, SE = .04) than grandiose symptoms (*M =* .00, SE = .00) as meeting BPD specifiers.

We used pairwise comparisons to examine the breakdown of the significant clinician gender × patient gender interaction. Only significant results are reported. Female clinicians rating female patients were more likely to perceive the patient as meeting borderline specifiers (*M =* .21, SE = .04) than male clinicians rating female patients (*M =* .00, SE = .00). Female clinicians rating male patients were more likely to perceive the patient as meeting borderline specifiers (*M =* .18, SE = .04) than male clinicians rating female patients (*M =* .00, SE = .00). Male clinicians rating female patients were less likely to perceive the patient as meeting borderline specifiers (*M =* .00, SE = .00) than male clinicians rating male patients (*M =* .23, SE = .07).

Pairwise comparisons were also used to break down the significant clinician gender × narcissism interaction. Female clinicians rated patients in grandiose vignettes (*M =* .13, SE = .03) as less likely to meet borderline specifiers than patients in vulnerable vignettes (*M =* .28, SE = .04) but more likely to meet borderline specifiers than male clinicians rating grandiose vignettes (*M =* .00, SE = .00). Female clinicians rated patients in vulnerable vignettes (*M =* .28, SE = .04) as more likely to meet borderline specifiers than male clinicians rating grandiose vignettes (*M =* .00, SE = .00). Male clinicians rated grandiose vignettes (*M =* .00, SE = .00) as less likely to display borderline specifiers than patients in vulnerable vignettes (*M =* .29, SE = .07).

A significant interaction between patient gender and narcissism was also significant and broken down using pairwise comparisons. Female patients in the grandiose vignette (*M =* .00, SE = .00) were less likely to meet borderline specifiers than female patients displaying vulnerable narcissism (*M =* .30, SE = .06) and compared with male patients presenting vulnerable narcissism (*M =* .28, SE = .05) and grandiose narcissism (*M =* .14, SE = .04).

## DISCUSSION

The purpose of the current study was to investigate the influence of gender bias on diagnostic ratings within the new ICD‐11 classification of personality disorders across diverse narcissistic expressions (grandiose/vulnerable) as presented in hypothetical male and female patient vignettes. The results presented overall suggest a potential ‘evidence of absence’ regarding gender bias within the new system. That is, for all domains of personality functioning (and overall personality severity) and the majority of personality trait domains, there was no significant variation in clinician ratings attributable the gender of the hypothetical narcissism vignette (or the gender of the rating clinicians). Instead, ratings varied substantially when comparing across narcissism subtype (grandiose/vulnerable).

While contrasting the rated personality severity and trait constellations of the narcissistic subtypes is outside the scope of the current paper (for more information, see Day et al., 2024), it is important to note as it indicates that clinicians anchored their ICD‐11 ratings based off relevant *clinical* material, and avoided obvious gender stereotype cues present in previous diagnostic systems. These findings are particularly relevant when compared to previous research conducted by Green et al. (2023) using a similar methodology and sample; however, in this prior study clinicians scored the hypothetical patients according to traditional categorical system of the DSM‐5. In contrast to the current study, Green et al. (2023) reported a significant influence of patient gender on clinician ratings with female hypothetical patients (but not male hypothetical patients) with vulnerable narcissism features diagnosed as BPD.

The relevance of the current research is also in light of the recent publication of the ICD‐11 whereby all categorical personality disorder ‘types’ have been removed, instead relying wholly on a personality functioning and trait model (Bach & First, 2018). While meaningful questions remain regarding acceptability and clinical utility of the new model due to the loss of accumulated theory and evidence regarding the previously established personality disorder types (Bach et al., 2022), our results suggest that a potential benefit of the new model is that it may force clinicians to attend to core self and interpersonal domains when making diagnoses, as opposed to relying on shorthand heuristic categories which may be laden with bias and stereotype (Braamhorst et al., 2015; Rienzi et al., 1995).

Having said that, while the ICD‐11 model was largely unaffected by gender bias, there are some discrepant findings that are important to outline. First, negative affectivity was the only trait domain that varied significantly across both participant and patient gender. That is, female patients were rated as displaying greater negative affectivity, with this being particularly true if the rating clinician was male. On the one hand, it can be argued that such gender bias is influenced by culturally prescribed gender stereotyped traits which depicts females as hypersensitive and neurotic (Ussher, 2017). On the other hand, these gender patterns align with self‐reported narcissism, upon which females endorse higher ratings on negative affectivity (e.g., Riegel et al., 2022). In other words, gender differences in reported trait domain of negative affectivity may reflect true phenomenological differences between men and women (Gomez et al., 2023; Suzuki et al., 2019). However, this interpretation of findings is tempered by the fact that in our sample vignettes were specifically made to contain identical symptomatology across genders, which indicates any discrepancies are more likely due to clinician bias. As such, it is important to acknowledge that clinicians should be cautious not to over‐diagnose or under‐diagnose certain trait manifestations in male and female patients due to potential gender stereotypes.

Second, gender bias also emerged in relation to the ICD‐11 ‘BPD specifier’. It is noteworthy that the specifier was not particularly endorsed by participants for either grandiose or vulnerable vignette; however, it was male patients who were more frequently endorsed as meeting BPD criteria, and this being particularly true if the rating clinician was female and the narcissism subtype was ‘vulnerable’. This gender bias is not what we would have expected, given the actual diagnostic rates in clinical practice of women being disproportionately over‐represented (Skodol & Bender, 2003). As such, this gender bias combined with the overall low endorsement rate may instead indicate the general spuriousness of the BPD specifier inclusion (Gutierrez et al., 2022) and reflect clinicians' diagnostic insecurity in relying on categorical ratings when they were unsure how else to conceptualize the presentation.

### Limitations and future directions

There are several limitations to consider to best contextualize the results of this study. First, utilizing a vignette‐based design is beneficial as it allows for easier systematic manipulation of variables and so increases confidence in the findings reported. However, the extent to which current findings can be generalized to actual clinician–patient interactions and diagnostic classifications is an area of future suggested research. Second, we utilized a shortened version of the PDS‐ICD‐11 that captured the core self and interpersonal domains, but not other elements (such as harm to self or others, reality testing, etc.). These modifications were made as the vignettes did not contain clear examples of these kinds of features within the description, so we focused on just the core features that were easier to be identified. It is possible that the items we removed from the survey may have picked up gender bias, particularly as the scoring would have been ambiguous (and so may have inferred more stereotype) due to not having clear examples within the vignettes.

Similarly, we used a brief and simple binary choice index of trait domains and facets for purposes of easing participant burden when completing the survey. While this does resemble how domains and traits are utilized in the ICD‐11, implementing a more extensive measure of personality traits may have resulted in more robust findings (Oltmanns, 2021). Suggested future research includes a need to replicate this study within clinical settings, to examine if the results hold within a naturalistic real‐world context regarding the presence (or absence) of gender bias when using the ICD‐11 classification system. Lastly, another limitation concerns the sampling technique used. Despite widely used in exploratory research due to its many strengths (cost effective, access to ‘hard‐to‐reach’ populations), snowball sampling tends to generate homogenous samples as individuals within the same networks share similar characteristics (Field, 2009). Future research should recruit a large diverse population to increase representativeness by using other sampling methods in addition to snowball sampling.

## CONCLUSION

The current study shows promising data for the clinical utility of the new ICD‐11 model in terms of capturing trait domains and severity of narcissistic grandiosity and vulnerability in male and female cases, largely absent of gender bias. Suggestions for future research include replicating our findings in more naturalistic settings, using the full measurements of the trait domains and severity indices to further disentangle the extent to which gender stereotypes in narcissistic presentations influence clinical assessment. More research is also needed to replicate the findings concerning the BPD specifier and further evaluate its clinical utility and inclusion in the ICD‐11 model.

## AUTHOR CONTRIBUTIONS

**A. Green:** Conceptualization; investigation; funding acquisition; methodology; writing – original draft; writing – review and editing. **N. J. S. Day:** Conceptualization; investigation; methodology; writing – review and editing. **C. M. Hart:** Software; formal analysis; data curation; writing – review and editing. **B. F. S. Grenyer:** Methodology; writing – review and editing. **B. Bach:** Methodology; writing – review and editing.

## CONFLICT OF INTEREST STATEMENT

The authors declare no conflicts of interest.

## Supporting information


Data S1.

## Data Availability

The data that support the findings of this study are available from the corresponding author upon reasonable request.
