# Supplementary material for: Gender bias in assessing narcissistic personality: Exploring the utility of the ICD‐11 dimensional model
Source: Br J Clin Psychol. 2024 Sep 11;64(2):248–64. doi: 10.1111/bjc.12503 (PMC12057326; doi:10.1111/bjc.12503)
Supplement: Supplementary file 1 — Data S1. [file BJC-64-248-s001.docx]

**Supplementary Results**

**Severity Items**

GEE models were run on each of the individual PDS items (see Table S1). A summary of findings is outlined below.

**PDS1 (identity).** The test of model effects revealed no significant main effects or interactions.

**PDS2 (self-esteem).** The test of model effects revealed only a significant effect of narcissism. Pairwise comparisons confirmed that clinicians rated patient symptoms as being more severe when presented with a patient with grandiose narcissism (*M =* 1.83, *SE =* .03) than vulnerable narcissism (*M =* 1.56, *SE =* .05).

**PDS3 (self-worth).** The test of model effects revealed only a significant effect of narcissism. Pairwise comparisons confirmed that clinicians rated patient symptoms as being more severe when presented with a patient with grandiose narcissism (*M =* 1.68, *SE =* .04) than vulnerable narcissism (*M =* 1.08, *SE =* .05).

**PDS4 (self-direction).** The test of model effects revealed only a significant effect of narcissism. Pairwise comparisons confirmed that clinicians rated patient symptoms as being more severe when presented with a patient with grandiose narcissism (*M =* 1.19, *SE =* .07) than vulnerable narcissism (*M =* 0.82, *SE =* .06).

**PDS5 (relationship interest).** The test of model effects revealed no significant main effects or interactions.

**PDS6 (Empathy).** The test of model effects revealed no significant main effects or interactions.

**PDS7 (Mutuality).** The test of model effects revealed a significant effect of narcissism. Pairwise comparisons confirmed that clinicians rated patient symptoms as being more severe when presented with a patient with grandiose narcissism (*M =* 1.71, *SE =* .04) than vulnerable narcissism (*M =* 1.16, *SE =* .06). There was also a significant clinician gender * narcissism interaction. Pairwise comparisons were used to break down this significant interaction. Female clinicians rated patients in grandiose vignettes (*M =* 1.62, *SE* = .05) as more severe on mutuality than patients in vulnerable vignettes (*M =* 1.26, *SE* = .06) and as more severe than male clinicians rating vulnerable vignettes (*M =* 1.07, *SE* = .11). Male clinicians rated grandiose vignettes (*M =* 1.80, *SE* = .07) as more severe than patients in vulnerable vignettes (*M =* 1.07, *SE* = .11) and as more severe than female clinicians rating vulnerable vignettes (*M =* 1.26, *SE* = .06).

**PDS8 (Conflict management)** The test of model effects revealed no significant main effects or interactions.

Table S1

*Test of Model Effects on Individual Severity Items*

|  | PDS1  (identity) | | PDS2  (self-esteem) | | PDS3  (self-worth) | | PDS4  (self-  direction) | | PDS5  (relationship interest) | | PDS6  (empathy) | | PDS7  (mutuality) | | PDS8  (conflict management) | |
| --- | --- | --- | --- | --- | --- | --- | --- | --- | --- | --- | --- | --- | --- | --- | --- | --- |
| Variables | Wald χ^2^ | *p* | Wald χ^2^ | *p* | Wald χ^2^ | *p* | Wald χ^2^ | *p* | Wald χ^2^ | *p* | Wald χ^2^ | *p* | Wald χ^2^ | *p* | Wald χ^2^ | *p* |
| (Intercept) | 4031.27 | <.001 | 6402.47 | <.001 | 5084.88 | <.001 | 1740.11 | <.001 | 1745.37 | <.001 | 4216.63 | <.001 | 3539.45 | <.001 | 4165.03 | <.001 |
| Clinician gender | .61 | .435 | .55 | .458 | .01 | .929 | 1.29 | .257 | .92 | .339 | .29 | .588 | .000 | .982 | .000 | .991 |
| Patient gender | .03 | .868 | 1.20 | .274 | .75 | .386 | .31 | .577 | .33 | .567 | .01 | .922 | .71 | .399 | .94 | .333 |
| Narcissism | .95 | .331 | 25.74 | <.001 | 86.81 | <.001 | 20.38 | <.001 | 1.32 | .250 | .18 | .675 | 69.95 | <.001 | .01 | .943 |
| Length of clinical practice | .23 | .631 | .34 | .560 | 1.24 | .266 | 1.59 | .207 | .76 | .383 | .03 | .860 | 1.00 | .318 | 1.32 | .251 |
| Clinician gender * Patient gender | .28 | .599 | .76 | .383 | .68 | .409 | 1.19 | .276 | .82 | .365 | .03 | .857 | 1.09 | .297 | .29 | .589 |
| Clinician gender * Narcissism | .45 | .504 | .04 | .834 | .61 | .437 | .003 | .958 | .09 | .766 | 2.26 | .132 | 7.99 | .005 | .73 | .394 |
| Patient gender * Narcissism | .37 | .054 | .89 | .346 | .90 | .344 | .02 | .896 | 1.31 | .253 | .65 | .419 | 1.55 | .213 | .01 | .939 |
| Clinician gender * Patient gender * Narcissism | 6.10 | .014 | .001 | .975 | 2.19 | .139 | .68 | .410 | .07 | .799 | 1.22 | .269 | 1.67 | .197 | 1.85 | .173 |

*Note*. Degrees of freedom = 1.
